# Decolonizing drug policy

**DOI:** 10.1186/s12954-021-00564-7

**Published:** 2021-11-27

**Authors:** Colleen Daniels, Aggrey Aluso, Naomi Burke-Shyne, Kojo Koram, Suchitra Rajagopalan, Imani Robinson, Shaun Shelly, Sam Shirley-Beavan, Tripti Tandon

**Affiliations:** 1 Harm Reduction International (HRI), 61 Mansell Street, Aldgate, London, E1 8AN UK; 2 Open Society Foundation, Open Society Initiative for Eastern Africa, ACS Plaza, 1st Floor, Lenana Road, P O Box 2193-00202, Nairobi, 00202 Kenya; 3 Birkbeck College, University of London, Malet St, London, WC1E 7HX UK; 4 Release, 61 Mansell Street, Aldgate, London, E1 8AN UK; 5 TB/HIV Care, South African Network of People Who Use Drugs (SANPUD), University of Pretoria, Department of Family Medicine, 7th Floor, 11 Adderley Street, Cape Town City Centre, Cape Town, 8001 South Africa; 6 Lawyers Collective, 4th floor, Jalaram Jyot, 63 Janmabhoomi Marg, Fort, Mumbai, 400001 India

**Keywords:** War on drugs, Drug control policy, Human rights

## Abstract

This paper reviews evidence of how drug control has been used to uphold colonial power structures in select countries. It demonstrates the racist and xenophobic impact of drug control policy and proposes a path to move beyond oppressive systems and structures. The ‘colonization of drug control’ refers to the use of drug control by states in Europe and America to advance and sustain the systematic exploitation of people, land and resources and the racialized hierarchies, which were established under colonial control and continue to dominate today. Globally, Black, Brown and Indigenous peoples are disproportionately targeted for drug law enforcement and face discrimination across the criminal system. These communities face higher arrest, prosecution and incarceration rates for drug offenses than other communities, such as majority populations, despite similar rates of drug use and selling among (and between) different races. Current drug policies have contributed to an increase in drug-related deaths, overdoses and sustained transnational criminal enterprises at the expense of the lives of people who use drugs, their families and greater society. This review provides further evidence of the need to reform the current system. It outlines a three-pillared approach to rebuilding drug policy in a way that supports health, dignity and human rights, consisting of: (1) the decriminalization of drugs and their use; (2) an end to the mass incarceration of people who use drugs; (3) the redirection of funding away from ineffective and punitive drug control and toward health and social programs.

## Introduction

“Only by dismantling unjust systems can we imagine a future that is safe, healthy, and truly free.”

- Colin Kaepernick, American civil rights activist and former footballer

The war on drugs has failed in its stated goal of reducing drug use. Instead, it has resulted in a devastating trail of trauma, pain and suffering for families and communities. Globally, it is people of color and other marginalized communities who have faced the harshest impact. Drug law enforcement has led to mass incarceration,^1^ arbitrary arrests and detention and devastating police brutality. Drug harms and drug-related deaths have increased the demand for unregulated drugs,^2^ and the high economic cost of punitive drug law enforcement has yet to show any return on investment.

Despite the failures of the war on drugs, a minority of countries has used their economic, political and military power to export their drug policies. Countries in the global south have been encouraged, coerced or obliged to criminalize (sometimes in the form of militarized interventions) responses to the drug trade and people who use drugs. Countries have been colonized by the insistence that they must combat the drugs trade and use of drugs through "universal action that calls for international cooperation”.^3^ This failed policy approach has created tools for, and served to sustain, the legacy of colonization.

This paper reviews evidence of how drug control has been used to uphold colonial power structures in select countries. It demonstrates the racist and xenophobic impact of drug control policy and proposes a path to move beyond oppressive systems and structures. For the purposes of this paper, *the colonization of drug control refers to the use of drug control by states in Europe and America to advance and sustain the systematic exploitation of people, land and resources, as well as racialized hierarchies, which were established under colonial control and continue to dominate today.* Calls for the decolonization of drug policy reflect the need to dismantle colonial power structures sustained by drug policy and to think beyond oppressive power structures, rather than a call to return to the precise legal or policy systems that were in place before colonization. This global war on drugs, rooted in racism and colonialism, must be replaced by strategies grounded in science, health and social equity and approaches that displace Western hegemonic systems of oppression.

## The colonial origins of contemporary drug prohibition

Human beings have used psychoactive substances for millennia. In Europe, the ancient Greeks used psychoactive substances prescribed by ancient physicians such as Hippocrates, Galen, and Ctesias to achieve a euphoric state of mind.^4^In the United Kingdom (UK) in the nineteenth century, the Victorians consumed alcohol, opium, cannabis, coca, mescaline and, following the invention of the hypodermic needle in the 1840s, morphine and heroin.^5^In pre-colonial Africa and much of Asia, cannabis was cultivated, traded and used as medicine.^6^ The plant has a sacred role in the Rastafarian, Sufi and Hindu religions. The Indigenous peoples of the Andean Amazon region revere the coca leaf. The opium poppy has a centuries-old history as traditional medicine and ceremonial use in Asia and the Middle East.^7^

Early in the European colonial project, psychoactive substances were among the commodities colonial powers traded and consumed. The commodities included sugar, tobacco, cannabis, opium and cocaine. In the nineteenth century, the Dutch planted coca plantations in Java, now Indonesia. The British interest in the opium trade was so great that it launched wars against the Ching dynasty in China for the right to sell the opium it was producing in the ‘East Indies.’ A British Royal Commission on Opium in 1895 reported no evidence of moral or physical degradation because of opium use.^8^ Similar conclusions were drawn in respect to cannabis use by the Indian Hemp Commission, 1894–1895.

However, just 66 years later, the Single Convention on Narcotic Drugs of 1961 was adopted at the United Nations Economic and Social Council. In less than a century, drugs had gone from being an essential part of European colonial trade to—in the words of the Single Convention—a “serious evil” that “leads to personal degradation and social disruption”.^9^

The rise of the United States of America (USA) as a colonial power in the early twentieth century was crucial to this transition. For example, when the Spanish handed over the Philippines, Guam and Cuba to the USA after the Spanish-American war in 1898, a project to ‘remake’ the native populations included controls on the use and trade of opium. At the Shanghai Opium Commission in 1909, the USA used its rising power to push the UK, France and other European powers to turn toward its vision. From 1909, there was a new era of drugs and the so-called civilizing mission of European colonialism. The expulsion of drugs was seen as a necessary element in turning ‘uncivilized’ people into the American vision of civilized, sovereign subjects.^10^

In parallel during this period, colonial legislation imposed oppressive, restrictive and punitive drug policies in Africa, rooted in pseudoscientific racism and concepts of moral responsibility. It hinged on Western political and religious doctrines that painted most African cultural practices as ‘evil’ and ‘backward.’ In Kenya, cannabis was banned by the colonial government under the 1933 Dangerous Drugs Act. Cannabis prohibition laid the foundations for the 1994 Narcotic Drugs and Psychotropic Substances (Control) Act, which governs Kenya’s drug policy today. In South Africa, the dismantling of apartheid in the 1990s led to the critical examination of a wide range of discriminatory and punitive policies. With the new South Africa came one of the world’s most progressive constitutions.^11^ However, some colonial ideas had been ‘made local’ and escaped scrutiny. Among the inherited narratives is the acceptance of the colonizers’ insistence that drugs are ‘evil.’ Today in South Africa, resources previously used to police apartheid have been shifted to the policing of drugs; policies and practices previously justified by apartheid became justified by the war on drugs.^12^ South Africa currently experiences a new form of colonization, with significant resources allocated to drug control by foreign powers, such as the US Drug Enforcement Agency and the Russian and Chinese governments.^13^

In India, colonial influence on drug policy was different but no less pernicious. The opium trade was heavily controlled and even promoted by the colonial state. In the early twentieth century, this led the Indian nationalist movement to criticize the role of opium in maintaining colonial rule.^14^ As a result, after independence in 1947, the post-colonial state and society appropriated prohibitionist ideas; the prohibitionist philosophy was even incorporated into the Indian Constitution, albeit as a non-binding principle.^15^ Today, the ‘war on drugs’ approach is so deeply entrenched that the country’s highest court has considered drug crimes “more heinous than murder*”*^16^—which is not only contrary to international human rights law^17^ but has also frustrated efforts to introduce proportionate sentencing for people convicted of drug-related offenses.^18^

The insistence in colonial and post-colonial narratives on ‘civilizing’ native populations, including their relationship with drugs, accelerated legal and political systems that permit state violence and harsh punishment for suspicion of drug use, cultivation or sale.^19^ Throughout the 20th and into the twenty-first century, the people who have suffered a heavy burden of the resulting counter-narcotic programs and violence are those in transit and producer areas, such as Latin America, the Horn of Africa and East Asia.^20^ In consumer countries, there is a racial ordering around who faces the heaviest weight of international drug prohibition.

## Post-colonial drug policy is racist and xenophobic

From its origins in the late nineteenth century until today, drug policy has been an instrument of repression and oppression inextricably tied to racism and xenophobia. Criminalizing and stigmatizing certain substances and making their use seem ‘deviant’ has served to demonize, dehumanize and marginalize the communities who use them. This strategy has been employed the world over to harm and repress ethnic minority groups, political dissidents, the poor and the dispossessed.^21^

Globally, Black, Brown and Indigenous peoples have been disproportionately targeted for drug law enforcement and face discrimination across the criminal system.^22^,^23^,^24^They face higher arrest, prosecution and incarceration rates for drug offenses than other communities, such as the majority population, despite similar rates of drug use and selling among (and between) different races.^25^,^26^

In 1994, John Ehrlichmann, a former Assistant for Domestic Affairs to US President Richard Nixon, vividly describes the opportunism of the Nixon administration’s new war on drugs:

The Nixon campaign in 1968, and the Nixon White House after that, had two enemies: the anti-war left and black people. We knew we couldn’t make it illegal to be either against the war or black, but by getting the public to associate the hippies with marijuana and blacks with heroin, and then criminalizing both heavily, we could disrupt those communities. We could arrest their leaders, raid their homes, break up their meetings, and vilify them night after night on the evening news. Did we know we were lying about the drugs? Of course, we did.^27^

The disproportionate policing of Black and Brown people persists to this day. Currently, in the USA, Black people are incarcerated at 5 times the rate of white people. Black people comprise 57 percent of all people incarcerated in state prisons, and 77 percent of people incarcerated in federal prisons for drug offenses are Black or Latino despite these populations making up just 30 percent of the US population.^28^

Around the world, punitive drug policy necessitates the surveillance, criminalization and targeting of Black, Brown and Indigenous people.^29^ In the UK, Black people are more than eight times more likely to be stopped and searched by police than white people.^30^, ^31^ In London during the COVID-19 pandemic, 20,000 young people of color were stopped and searched by police, the equivalent of more than a quarter of all black 15 to 24-year-olds in London. More than 80 percent of the 21,950 searches between March and May resulted in no further action.^32^,^33^In 2018, in Rio de Janeiro, Brazil, 80 percent of those killed by police were Black.^34^ Sixty-four percent of Brazilian prisoners are Black, and 65 percent of those convicted are imprisoned for drug offenses.^35^These patterns are replicated worldwide and have been recognized by the UN Working Group of Experts on People of African Descent through the following statement:

The war on drugs has operated more effectively as a system of racial control than as a mechanism for combating the use and trafficking of narcotics. … [It] has disproportionately targeted people of African descent and disregarded the massive costs to the dignity, humanity and freedom of individuals.^36^

The global drug control regime has also undermined the rights of Indigenous peoples. This includes a similar disproportionate policing response to drug use in Indigenous communities as that experienced by Black and Brown people. In Australia, Indigenous people are 15 to 20 times more likely to be incarcerated than non-Indigenous people.^37^,^38^In Canada, criminal law continues to disproportionately harm Black and Indigenous communities.^39^ Indigenous communities also experience violations of their rights where cultural traditions are limited by drug control. The 1961 Single Convention on Narcotic Drugs, grounded in the colonial denial of non-Western systems of knowledge, obliges all states to abolish the traditional use of coca, cannabis and opium through crop eradication and drug law enforcement. The conflict between the drug control regime and Indigenous rights continues today. Crop eradication campaigns have militarized coca-producing areas and displaced Indigenous people to neighboring countries in the Andes,^40^ and anti-drug rhetoric has been used in Brazil to justify police raids in Black and Indigenous communities.^41^

## Decolonizing drug policy: decriminalize, decarcerate, divest and redirect

Current drug policies have contributed to an increase in drug-related deaths, overdoses and sustained transnational criminal enterprises at the expense of the lives of people who use drugs, their families and greater society. Reforming this system requires not just tinkering with the current approach but interrupting these systems of violence at their root.

The effort to rebuild drug policy in a way that supports health, dignity and human rights must have three pillars: the decriminalization of drugs and their use; an end to the mass incarceration of people who use drugs; and the redirection of funding away from ineffective and punitive drug control towards health and social programs.

### Decriminalize: remove criminal and administrative penalties for drugs and drug use

Decriminalization is the central building block on which a new drug policy can be built. It would eliminate all criminal and administrative penalties, reduce the number of people in prison and prioritize health and safety over punishment for drug use. Experiences of decriminalization to date have demonstrated its role in reducing the adverse health, social and economic impact of drug policy on people who use drugs and society as a whole. The International Network of People who Use Drugs calls for:

All models of decriminalization [to] **fully decriminalize** people who use drugs, including: the removal of all administrative sanctions and mechanisms of monitoring, surveillance, coercion and punishment for use and possession of drugs; removing the use of arbitrary quantity thresholds or threshold amounts that result in criminal records; ensuring that operational police fully understand policy and legislative changes associated with full decriminalization; and establishing independent and ongoing monitoring for criminal justice systems.^42^

In 2001, Portugal enacted one of the most extensive drug law reforms by decriminalizing the personal use and possession of *all* illicit drugs, while retaining criminal sanctions for activities such as trafficking. The Portuguese Government focused efforts on treatment and harm reduction. The change in policy resulted in no significant increase in rates of drug use but did lead to a fall in new HIV infections among people who inject drugs (from 1,575 in 2000 to just 78 in 2013), and a fall in drug‐induced mortality, from 80 deaths in 2001 to just 16 in 2012.^43^

The United Nations System Common Position on Drug Policy (2018) commits to supporting Member States in implementing truly balanced, comprehensive, integrated, evidence-based, human rights-based, development-oriented, sustainable responses to the world’s response to drugs. It calls for a rebalancing of drug policies toward health and human rights and promotes “alternatives to conviction and punishment and [to] consider shifting to a non-punitive, regulatory framework that prioritizes public health, equity and social justice in drug control. This includes the decriminalization of drug possession for personal use.”^44^

## Decarcerate: reduce prison population globally

In many countries, governments use drug laws to disproportionately criminalize people associated with a particular race or ethnicity. The war on drugs provides states with a tool to justify the social control of minorities and marginalized communities. Decriminalization must be accompanied by decarceration and releasing people held in custody or in prisons because of drug offenses.

Since 2000, the world prison population has grown by 20 percent.^45^ The female prison population has increased by 50 percent.^46^ Over 11 million people are imprisoned worldwide today, the highest number ever recorded.^47^ Punitive drug policies and laws continue to drive this mass incarceration: 1 in 5 people in prison globally—2.5 million people—are detained because of drug offenses,^48^ and the proportion is even higher among women.^49^, ^50^UNAIDS estimates that 56–90 percent of people who inject drugs will be incarcerated at some stage in their lifetime.^51^In 1980, 580,900 people were arrested on drug‐related charges in the USA. By 2014, that number had increased to 1.56 million. Nearly half of the 186,000 people serving time in federal prisons in the USA are incarcerated on drug‐related charges.

Black, Brown, and Indigenous people are overrepresented in the world’s prisons. Higher arrest and incarceration rates for these communities do not reflect a higher prevalence of drug use; rather they reflect law enforcement’s greater focus and greater use of violence and force in urban areas, lower-income communities and communities of color.^52^

The consequences of incarceration can transcend individuals and even generations. Incarceration of a parent or breadwinner can impact a family’s income and ability to fulfill its basic needs. The negative consequences of incarceration are more severe and long lasting for women—impacting health, finances, social stability, family and personal relationships. Negative consequences for children can extend to social exclusion, educational attainment, housing status and health.^53^,^54^, ^55^ These effects are compounded in the social groups that are more likely to experience incarceration, reinforcing pre-existing inequalities related to race, nationality and class.

For those incarcerated for drug offenses, the negative consequences can extend far beyond prison, as drug offenses can impact individuals for years or even a lifetime. In the USA, for example, people are penalized throughout their working careers, as a criminal record can severely limit job opportunities.^56^In the UK, benefit payments and entitlements may stop or change if a person, their partner or child is sent to prison or is in custody awaiting trial.^57^ This has particularly strong implications for ethnic minorities and other historically disadvantaged groups. In the USA, students are denied financial aid due to drug convictions.^58^ Efforts to decolonize drug policy and combat racial inequality must also create opportunities to heal and restore past harms and injustice. As the recent cases of Breonna Taylor and George Floyd in the USA and Rashan Charles in the UK exemplify, the war on drugs is used to justify the callousness with which Black lives are taken.^59^

Drug policy reform must be driven by the transformation of our relationship to punishment, which is to say a radical commitment to a new world, or it risks no meaningful change in the long term.^60^ States must support people to live healthy lives by not solely relying on the prison system as a catch-all solution to social ills,^61^ and by creating, developing, practicing and promoting alternatives to incarceration. In 2020, many drug policy reformers with an anti-racist and decolonial framework for reform supported demands from prison abolitionists to divest from law enforcement entirely. This calls for a drug policy landscape that is able to operate entirely separately from the carceral state, that is, without policing, prisons, surveillance and coercion (e.g., forced abstinence or treatment). Abolishing the prison system and the carceral state arguably extends the widely valued harm reduction philosophy to both ‘do no harm’ and to actively support, not punish people who use drugs.^62^

### Divest and redirect: move funding from ineffective law enforcement to essential harm reduction and other social and community programs

Every year, USD 100 billion is spent on global drug law enforcement, roughly 750 times more than the amount invested in life-saving services for people who use drugs.^63^To decolonize drug policy, funds must be redirected away from the institutions that uphold racist, discriminatory policies and disrupt the white supremacist system created in service of colonial violence.^64^ Calls for funding to be redirected from ineffective, punitive drug law enforcement to social, health and other community services must be heeded if drug policy reform is to address the root causes of the harms created by the war on drugs.^65^

In 2019, the total budget for harm reduction in Thailand was estimated to be USD 1.7 million; in contrast, the Thai government allocated around 1,500 times this amount to drug law enforcement activities.^66^Drug law enforcement expenditure in Thailand is USD 1.8 billion. In Indonesia, drug law enforcement is estimated to be USD 250 million, of which USD 81 million is for prison costs for drug-related offenses and USD 31 million for prison costs for possession for personal use. The Drug Enforcement Administration in the USA, which cost US tax-payers USD 3.136 billion^67^ in the financial year 2019, is an organization that militarizes police in the USA as well as other countries, such as South Africa, to enforce drug control policy.

Harm reduction interventions that seek to reduce the negative health and social harms of drug use and drug policy are drastically under-implemented and underfunded. Fewer than half of the 179 countries where injection drug use occurs implement needle and syringe programs (NSPs). Even in those countries that do, coverage is generally low and limited to certain regions and urban centers.^68^ There are also considerable differences between the regions in terms of harm reduction implementation. While NSPs are available in most countries in Eurasia, North America and Western Europe, they are severely lacking in the majority of countries in other regions. An unfavorable drug policy environment hinders harm reduction service implementation in many countries across Asia, Latin America, the Caribbean, the Middle East and Africa.^69^ For people in prison, the situation is even starker: only 11 countries around the world have NSPs in prison.^70^

The solution can be simple: redirect resources from the billions spent on drug control to fund harm reduction and other health and social services for the people impacted by drug policy.

In Austin, Texas, USA, the city council redirected USD 150 million in funds from law enforcement to purchase housing for people experiencing homelessness, and to expand healthcare, access to food and prevent violence. In New York City, USA, there are plans to redistribute USD 1 billion to youth, education and other social services.^71^ In Denver, Colorado, USA, the city has been running a program to send medics and clinicians instead of the police out on emergency calls related to mental health, homelessness and substance use. As a result, people in crisis in Denver received help without having to talk to police on 748 occasions. No one was arrested, and people received healthcare and opportunities to heal instead.^72^ These are some examples of models that show how we can change the system.

In addition to a redirection of resources, reforming drug policy requires a relocation of influence. In the context of the aims of decolonizing drug policy, it is unsustainable that the UN agency tasked with dealing with crime, the UN Office on Drugs and Crime (UNODC), also holds the portfolio on drug use, which is an issue of health and bodily autonomy. With a broader mission of making the world safer from drugs, crime, corruption and terrorism, and an active commitment to supporting governments in the practical implementation of the colonial international drug policy commitments,^73^ there is little space for questioning the hegemonic powers behind international drug policy or creating political support to explore a decolonized path forward. A clear demonstration of this tension is found in the UNODC strategy, which seeks to improve HIV prevention, treatment and care for people who use drugs but fails to use the term ‘harm reduction.’ Focusing on prevention, treatment and care allows UNODC to endorse services rather than engaging with the more holistic approach to drug use inherent in the term harm reduction. As described by Harm Reduction International:

Harm reduction is rooted in a commitment to addressing discrimination and ensuring that nobody is excluded from the health and social services they may need because of their drug use, their race, their gender, their gender identity, their sexual orientation, their choice of work, or their economic status. People should be able to access services without having to overcome unnecessary barriers, including burdensome, discriminatory regulations.^74^

Similarly, UNODC’s mission sees its law enforcement and drug control operations conflict with, and impede on, the rights of people who use drugs and other vulnerable groups. In 2020, UNODC announced funding to support the refurbishment of a specialized voluntary drug rehabilitation center in Sri Lanka,^75^ even though there have been extensive reports of human rights violations, abuse and ill-treatment in drug detention facilities in Sri Lanka.^76^ Between 2012 and 2014, human rights groups called for UNODC to end its support for counter-narcotics police operations in Iran, recognizing UNODC resources were fueling police processes that resulted in the use of the death penalty for drug offenses, in violation of international human rights law.^77^

The 1961 Single Convention on Narcotic Drugs and the two subsequent treaties in 1971 and 1988 have been deployed to consolidate trade gains and secure the supremacy of colonial powers. We need to halt ongoing programs that fuel human rights violations in the name of drug control, such as the death penalty, compulsory detention and rehabilitation, and interrogate ongoing investments in these systems, including the role of state funding and UN actors, as part of efforts to divest and redirect.

## Conclusion

To combat the perpetuation of colonial power through international drug control conventions and the resulting prohibitionist drug policy, we must connect with other social justice movements to challenge oppressive systems and dismantle mutually reinforcing destructive policies. International consensus around drug control is broken and it will be important to replace it, not with States developing separate policies that sustain colonial ideologies, but with an entirely new approach grounded in human rights, social justice and science. Only an approach that challenges racialized hierarchies and recognizes the many systems of knowledge around the world can shift us beyond the systems of control and oppression currently upheld by drug control.

Decriminalization, decarceration, divestment and redirection are key. We must demand societies where it is inconceivable that any system upholds and justifies racism or colonial power structures. The dignity, autonomy and agency of Black, Brown and Indigenous communities—the global majority—must be at the center of all efforts.

## Abbreviations

HRI: Harm Reduction International
NSP: Needle and syringe programs
SANPUD: South African Network of People Who Use Drugs
UNODC: United Nations Office on Drugs and Crime
UNAIDS: Joint United Nations Programme on HIV/AIDS

## Footnotes

1 Among others, see: Alexander [1], Kerr and Jackson[2], Cooper [3].

2 UNODC (2021), ‘World Drug Report 2021’ [pdf].

3 United Nations General Assembly, Single Convention on Narcotic Drugs of 1961, as amended by the 1972 Protocol.

4 Laios et al. [4].

5 Crane, L. / Wellcome Collection (28 April, 2011), ‘Drugs in Victorian Britain’ [web article, accessed October 2021].

6 Duvall, CS. (2019), ‘A brief agricultural history of cannabis in Africa, from prehistory to canna-colony,’ EchoGéo, 48.

7 Kritikos, PG. and Papadaki, SP. / UNODC (1967), ‘The History Of The Poppy And Of Opium And Their Expansion In Antiquity In The Eastern Mediterranean Area’ [web resource, accessed October 2021].

8 Royal Commission on Opium Great Britain (1895), ‘Final Report of the Royal Commission on Opium Great Britain, Sessional Papers of the House of Commons,’ XLII, p.124.

9 Hobson [5], Lines [6].

10 Musto [7], Wright [8].

11 Cameron [9].

12 Scheibe et al. [10].

13 Shelly and Howell [11].

14 Framke [12].

15 Tandon, T. / IDPC (2015), ‘Drug policy in India. International Drug Policy Consortium, Briefing Paper’ [pdf].

16 See Union of India v Ram Samujh (1999), 9 SCC 429.

17 Lines [13].

18 See Gurdev Singh v State of Punjab (Date unknown), SCC OnLine SC 28.

19 Koram [14].

20 Ibid.

21 Amnesty International (4 February, 2017), ‘Philippines: Duterte's 'war on drugs' is a war on the poor’ [web article, accessed October 2021].

22 Rahamatulla, A. and Ford Foundation (23 March, 2017), ‘The War on Drugs has failed. What’s next?’ [web article, accessed October 2021].

23 Koram [14].

24 Burke-Shyne, N. and Daniels, C. / Talking Drugs (4 June, 2020), ‘Racism and Policing are Global Problems, Drug Control is a Vehicle’ [web article, accessed October 2021].

25 Drug Policy Alliance (2015), ‘The Drug War, Mass Incarceration and Race’ [pdf].

26 Release, StopWatch and LSE (2020), ‘The Colour of Injustice: ‘Race,’ drugs and law enforcement in England and Wales’ [pdf].

27 Drug Policy Alliance, ‘A Brief History of the Drug War’ [web page, accessed October 2021].

28 NAACP, ‘Criminal Justice Fact Sheet’ [web page, accessed October 2021].

29 OHCHR (14 March, 2019), ‘Fight against world drug problem must address unjust impact on people of African descent, say UN experts’ [media statement, accessed October 2021].

30 Gov.UK (22 February, 2021), ‘Stop and search’ [web page, accessed October 2021].

31 Townsend, M. / The Guardian (4 May, 2019), ‘Black people’40 times more likely’ to be stopped and searched in UK’ [web article, accessed October 2021].

32 Grierson, J. / The Guardian (8 July, 2020), ‘Met carried out 22,000 searches on young black men during lockdown’ [web article, accessed October 2021].

33 Gov.UK (22 February, 2021), ‘Stop and search’ [web page, accessed October 2021].

34 Soares, J. / DW.com (6 October, 2020), ‘Racist police violence endures in Jair Bolsonaro's Brazil’ [web article, accessed October 2021].

35 Connectas Human Rights (8 December, 2017), ‘Brazil has the world’s 3rd largest prison population’ [web article, accessed October 2021].

36 OHCHR (14 March, 2019), ‘Fight against world drug problem must address unjust impact on people of African descent, say UN experts’ [media statement, accessed October 2021].

37 Australian Human Rights Commission, ‘Indigenous Deaths in Custody: Arrest, Imprisonment and Most Serious Offence’ [web page, accessed October 2021].

38 Australian Law Reform Commission (9 January, 2018), ‘Disproportionate incarceration rate’ [web page, accessed October 2021].

39 Government of Canada, Office of the Correctional Investigator (21 January, 2020), ‘Indigenous People in Federal Custody Surpasses 30%: Correctional Investigator Issues Statement and Challenge’ [news release, accessed October 2021].

40 IDPC, ‘Protecting the rights of indigenous people’ [pdf].

41 Henman [15].

42 INPUD (2021), ‘Drug Decriminalisation: Progress or Political Red Herring?’ [pdf].

43 Transform Drug Policy (13 May, 2021), ‘Drug Decriminalisation in Portugal: Setting the Record Straight’ [web article, accessed October 2021].

44 UN System Chief Executives Board for Coordination (2018), ‘Summary of deliberations: Chief Executives Board for Coordination, 2nd regular session of 2018, New York, 7 and 8 November 2018’ [pdf].

45 Walmsley, R. / World Prison Brief (2015), ‘World Prison Populations List’ [pdf].

46 Ibid.

47 Penal Reform International (2020), ‘Global Prison Trends 2020’ [pdf].

48 UNODC (2021), ‘World Drug Report 2021’ [pdf].

49 Penal Reform International (2015), ‘Global Prison Trends 2015’ [pdf].

50 UNODC (2018), ‘World Drug Report 2018: Women and Drugs’ [pdf].

51 UNAIDS (2014), ‘GAP Report 2014: People left behind: People who inject drugs’ [pdf].

52 Drug Policy Alliance, ‘Race and the Drug War’ [web page, accessed October 2021].

53 Gatti et al. [16], Gilman [17].

54 Gilman et al. [18].

55 Doherty et al. [19].

56 Couloute, L. and Kopf, D. / Prison Policy Initiative (July 2018), ‘Out of Prison & Out of Work: Unemployment among formerly incarcerated people' [pdf].

57 Gov.UK, ‘Benefits and Prisons’ [web page, accessed October 2021].

58 ACLU, ‘Injustice 101: Higher Education Act Denies Financial Aid To Students With Drug Convictions’ [web page, accessed October 2021].

59 Robinson, I. / Talking Drugs (29 May, 2020), ‘The War on Drugs and the Justification of Black Death’ [web article, accessed October 2021].

60 Robinson, I. / Talking Drugs (31 May, 2019), ‘Towards an Abolitionist Drug Policy Reform’ [web article, accessed October 2021].

61 Davis [20].

62 Robinson [21].

63 Transform Drug Policy Foundation (2013), ‘The War on Drugs: Wasting Billions and Undermining Economies’ [pdf].

64 Harm Reduction International (2018), ‘The lost decade: Neglect for harm reduction funding and the health crisis among people who use drugs’ [pdf].

65 Harm Reduction International (2021), ‘Failure to Fund: The Continued Crisis For Harm Reduction Funding In Low- And Middle-Income Countries’ [pdf].

66 Tanguay [22].

67 Drug Enforcement Administration, ‘Staff and Budgeting’ [web page, accessed October 2021].

68 Harm Reduction International (2020), ‘Global State of Harm Reduction 2020’ [pdf].

69 Ibid.

70 Ibid.

71 Collins, S. / The Vox (23 September, 2020), ‘The Financial Case For Defunding The Police’ [web article, accessed October 2021].

72 Schmelzer, A. / The Denver Post (7 September, 2020), ‘Call Police for a Woman Who is Changing Clothes in an Alley? A New Program in Denver Sends Mental Health Professionals Instead’ [web article, accessed October 2021].

73 UNODC (2021), ‘Strategy 2021–2025’ [pdf].

74 Harm Reduction International, ‘What is harm reduction?’ [web page, accessed October 2021].

75 UNODC (26 June, 2020), ‘Sri Lanka: Ground-breaking of new voluntary drug rehabilitation centre’ [web article, accessed October 2021].

76 Satkunanathan, A. / Harm Reduction International (2021), ‘Drug Control, Detention and Treatment of People Who Use Drugs in Sri Lanka’ [pdf].

77 Human Rights Watch (17 December, 2014) ‘UN: Freeze Funding Iran Counter Narcotics’ [web article, accessed October 2021]; Gallahue, P., Saucier, R. and Barrett, D. / Harm Reduction International (2012), ‘Partners in Crime, International Funding for Drug Control and Gross Violations of Human Rights’ [pdf].
